# Vegan Diet Health Benefits in Metabolic Syndrome

**DOI:** 10.3390/nu13030817

**Published:** 2021-03-02

**Authors:** Giulia Marrone, Cristina Guerriero, Daniela Palazzetti, Paolo Lido, Alessandro Marolla, Francesca Di Daniele, Annalisa Noce

**Affiliations:** 1 UOC of Internal Medicine-Center of Hypertension and Nephrology Unit, Department of Systems Medicine, University of Rome Tor Vergata, Via Montpellier 1, 00133 Rome, Italy; cristinaguerriero@hotmail.it (C.G.); daniela.palazzetti96@gmail.com (D.P.); paulshore@virgilio.it (P.L.); francesca.didaniele@gmail.com (F.D.D.); 2 PhD School of Applied Medical, Surgical Sciences, University of Rome Tor Vergata, Via Montpellier 1, 00133 Rome, Italy; 3 School of Specialization in Geriatrics, University of Rome Tor Vergata, 00133 Rome, Italy; alessandromarolla91@gmail.com

**Keywords:** plant-based diet, vegan diet, dietary pattern, metabolic syndrome, cardiovascular disease, chronic non-communicable diseases

## Abstract

Plant-based diets (PBDs) are increasingly consumed by the Italian population and around the world. In particular, among PBDs, the vegan diet is a food pattern characterized by the exclusion of all animal-origin foods. What drives people to adopt this model are mainly ethical, health and environmental reasons. A vegan diet, if well-balanced and varied, can help in achieving and maintaining an optimal state of health. However, this nutritional approach, if not well-balanced, can cause deficiencies in proteins, ω-3 fatty acids, iron, vitamin D and calcium, zinc, iodine and, above all, vitamin B12. Oral food supplements especially fortified foods are recommended in these cases to restore the nutritional deficiencies. A vegan diet generally reduces the risk of developing chronic non-communicable degenerative diseases, such as metabolic syndrome (MetS) and, in addition, requires fewer natural resources for food production than an omnivorous diet. The aim of this review is to analyze the possible impact of the vegan diet on MetS onset and its treatment.

## 1. Introduction

**Figure 1:**
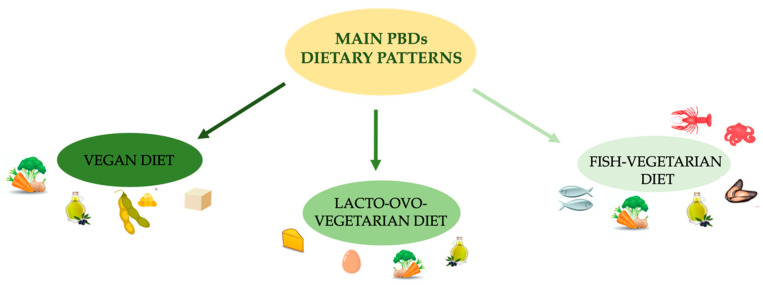
Main PBD dietary patterns. Abbreviation: PBDs, plant-based diets.

The most commonly adopted eating patterns in Western countries are represented by the Mediterranean diet (MD), dietary approaches to stop hypertension (DASH), paleolithic diet, low-carb and low-fat diets, and plant-based diet (PBD) [1,2,3,4,5] (Figure 1).

Over the past few years, the interest in PBD has increased both in the general population and in the scientific community, until it has become one of the main dietary patterns adopted in Western countries. In fact, PBDs have undergone a notable spread thanks to the ever-increasing number of people who have adopted it [6].

Among PBDs, it is possible to distinguish different types of diet, in particular, diets that exclude meat and fish but allow the consumption of milk, dairy products and eggs and diets which eliminate any animal-origin food [7,8]. The latest evidence suggests that PBDs are associated with a significant reduction in the risk of cardiovascular disease (CVD) and cancer onset [9]. Subjects following a PBD usually have a lower body mass index (BMI), and reduced total cholesterol (TC), low-density lipoprotein cholesterol (LDL-C), triglycerides (TG) and blood glucose levels compared to omnivores [9,10,11,12,13,14].

The main concern regarding these dietary approaches is the risk of developing nutritional deficiencies of proteins, ω-3 fatty acids, vitamin B12, iron, zinc, iodine, vitamin D and calcium. However, PBDs are rich in fruit and vegetables, and they are characterized by a high content of fiber, antioxidant substances, phyto-chemicals and ω-6 fatty acids [15]. If well planned and balanced, PBDs can be appropriate for all age groups, even in pregnancy or breastfeeding women [16].

People who voluntarily choose to adopt a PBD have an increased interest in the environment and in the animal world for ethical, health and/or economic reasons [17].

While they are several clinical trials supporting PBD, information on the vegan diet, provided by long term clinical studies, us scarce [18]. The aim of this review is to collect and assemble recent available data in the literature to investigate whether the vegan diet is useful for good health maintenance [18]. In fact, if properly balanced, the vegan diet can be a valid therapeutic adjuvant tool to counteract metabolic syndrome (MetS) onset and to reduce CVD risk [7,9,19,20]. In this review, we discuss the potential benefits arising from the adoption of a vegan diet compared to an omnivorous diet, in order to prevent and/or treat MetS and CVD. In particular, we explored the effects induced by PBDs, focusing on vegan diet (where available) and on its impact on the clinical impairments typical of MetS, such as high blood pressure values, high body weight and body circumferences, dyslipidemia and glucose intolerance. Moreover, we discuss the positive effects exerted by these dietary patterns on CVD risk.

## 2. Search Results

**Figure 2:**
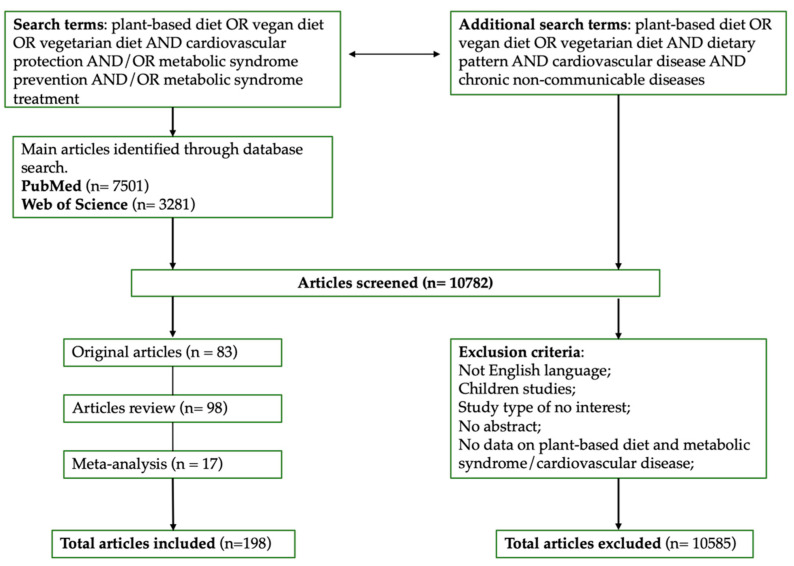
Full search strategy.

All the studies selected looked at the relationship between index terms “plant-based diet” or “vegetarian diet” or “vegan diet” and “cardiovascular protection” and/or “metabolic syndrome prevention” and/or “metabolic syndrome treatment”. The full search strategy is reported in Figure 2. The databases used were PubMed and Web of Science up to January 2021. Studies were all in the English language and references for the included studies were manually retrieved.

## 3. Plant-Based Dietary Patterns and Environmental Impacts

In recent years, PBDs have been growing all over the world. The origins of this “new food model” date back to the 6th century B.C., when some religious movements adopted a meat-free diet. In general, in the field of PBD nutrition, it is possible to distinguish three main models, which are the most commonly studied in literature, namely (i) lacto-ovo-vegetarian (LOV), (ii) vegan and (iii) fish-vegetarian (FV) patterns (Table 1).

In particular, the LOV model excludes all types of meat, but it includes cheese and various dairy products, eggs, honey and all vegetable-origin foods [21]. The vegan model eliminates all types of meat, milk and dairy products [22], eggs and honey, while the FV model excludes all types of meat, except fish and seafoods [8,23].

In a study conducted by Tonstad et al., the authors showed that the LOV diet was associated with a statistically significant reduction of type 2 diabetes mellitus (T2DM) incidence and it was protective for both black and non-black subjects, compared to omnivores [24]. Furthermore, it has been shown by Pettersen et al. that subjects who followed a LOV diet display lower systolic blood pressure (SBP) and diastolic blood pressure (DBP) values than omnivores [25].

The FV diet was analyzed in the study conducted by Clarys et al., who compared different dietary patterns. The results showed that subjects who followed a FV diet had a lower BMI and total energy intake, and a better nutritional quality than omnivores. The same authors demonstrated that, among PBDs, vegans showed even better results in terms of body composition, energy intake and food quality compared to FV [8]. Furthermore, it was highlighted by Petermann-Rocha et al. that subjects following a FV diet have a reduced risk of developing CVDs like myocardial ischemia, stroke and heart failure compared to omnivores [26]. In fact, fish and other seafoods are rich in ω-3 fatty acids, group B vitamins, zinc, iodine, selenium, calcium and magnesium, which are fundamental cardioprotective elements [27,28]. PBDs are classified according to the quality and the frequency of animal-origin food intake, rather than by the quality and the frequency of vegetable-origin food consumption. However, not all plant food is the same and there is a wide variety of PBDs, each with peculiar cardiometabolic effects [29,30,31]. Moreover, it should be noted that the term “PBD” does not necessarily mean “healthy”, since there is evidence that certain non-animal-origin food such as refined cereals, snacks, pastries or sugary drinks are harmful to health [32]. PBD to be defined as “healthy” must promote the intake of whole grains, fruit, vegetables, legumes and non-hydrogenated vegetable oils, such as extra-virgin olive oil (EVOO) [33,34,35]. Finally, the substitution of certain animal-origin food helps in reducing the intake of harmful components present in red and processed meats, such as excessive sodium, heme iron, nitrates and nitrites, which increase the risk of CVDs and MetS onset [33].

The Dietary Guidelines Advisory Committee (DGAC) in 2015 analyzed the link between food models and environmental impact by saying: “Consistent evidence indicates that, in general, a dietary model that is higher in PBD such as vegetables, fruits, whole grains, legumes, nuts and seeds, and lower in food of animal origin promotes more health and is associated with a lower environmental impact (greenhouse gases and energy consumption, soil and water) than the current average US diet” [36]. In support, the DGCA, evidence shows that dietary patterns that promote health also improve the environmental impact and, among these are MD, PBD, vegan and other types of diet [37,38,39,40,41,42,43,44].

It is known that the ability to produce enough food in the future is potentially limited by the availability of water, fertile land, and by the use and the management of the seas and oceans. Future generations could run out of natural resources [45] if energy, water and soil are not managed and stored responsibly [46]. In addition, food production contributes significantly to biodiversity loss; moreover, world population growth, increased energy costs and climate changes will continue to undermine available natural resources [34,47]. Several studies of various dietary patterns demonstrated that diets with reduced meat consumption, in particular PBDs and vegan diet, have yielded better results in terms of health status, reduction of greenhouse gas (GHG) emissions and land, energy and water use [48,49,50]. In the study conducted by Tilman et al., PBDs were compared to an omnivorous diet by analyzing the 100 most populous nations. The study found that vegan diet decreased mortality for all causes compared to omnivorous diet [51]. In addition, the comparison of these dietary patterns predicted by 2050 a reduction in GHG emissions and land use for the vegan diet compared to the omnivorous diet [52].

Another aspect that characterizes the vegan diet is linked to the absence of antibiotic residues coming from fodder that is provided to the animals in intensive farming [53,54]. Thus, the vegan diet allows the maintenance of optimal gut microbiota composition, favoring the state health [55,56,57].

### Strength and Weakness of Vegan Diets

**Figure 3:**
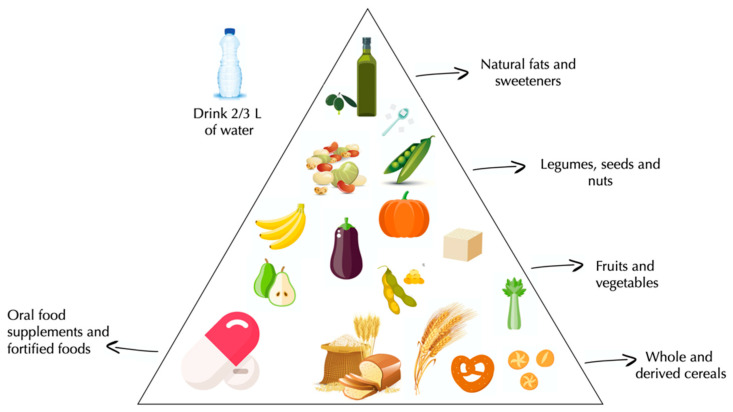
Vegan pyramid.

Today, the world prevalence of vegan diet varies from 2% of Americans, to less than 1% of Germans, to 2.2% of Italians [58,59]. Vegan diet is characterized by a high intake of whole and derived cereals, fruit, vegetables, legumes, seeds, nuts and vegetable oil (Figure 3).

People often adopt these food models for health reasons, since scientific evidence shows that vegan diet is able to promote, or to restore, “good health”, in particular affecting variables such as body weight or blood pressure (BP) [60,61].

Although the vegan diet represents a “healthy model”, it should nevertheless provide the correct intake of nutrients. In fact, one of the vegan diet’s potential “limits” is represented by the possible nutritional deficiencies that may arise due to the restriction of all animal-origin food [62]. Otherwise, these dietary patterns could present deficiencies, defined as a qualitative or quantitative lack of nutrients or vitamins, able to impair the physiological functions in the human organism [63]. For this reason, a vegan diet should be well-planned and supervised by a nutritionist, in order to avoid possible nutritional deficiencies [64].

Vegan diet is generally rich in carbohydrates, ω-6 fatty acids, dietary fibers, carotenoids, folic acid, vitamin C, vitamin E and magnesium and relatively low in proteins, ω-3 fatty acids, vitamin B12, vitamin D and calcium, iron, zinc and iodine [62,65,66].

The protein intake in the vegan diet is guaranteed by the combination of legumes and cereals [67]. Current food technologies have made it possible to develop plant-origin food similar to that of animal origin, such as the use of soy and its derivatives, which allows an adequate protein intake that otherwise could be lacking [68,69].

Numerous studies have tried to assess whether a vegan regime can provide an adequate protein intake [8,70,71,72], and most of these state that the average protein intake was 13–14% of daily caloric intake, thus representing an adequate value according to the American Guidelines [73]. A study conducted by Alles et al. analyzed the data obtained from the “Nutrinet-Sante Study” stating that 27% of vegans do not reach 10% of daily protein intake, questioning the adequacy of protein intake among vegans [74]. For this reason, the debate about the adequacy of protein intake remains unsolved.

Several studies conducted on ω-3 fatty acids’ plasma levels have shown that the vegan diet induces lower values of eicosapentaenoic acid (EPA) and docosahexaenoic acid (DHA). In particular, vegans have reduced levels of these fatty acids, compared with LOVs [75,76]. In fact, PBDs are generally rich in linoleic acid (e.g., flaxseed/linseed oils, chia seeds, flaxseed raw) that is able to reduce the conversion of α-linolenic acid (ALA) to EPA and DHA [77,78]. Other authors speculate that in vegans the mean ALA intake is at, or close, to an adequate intake (AI), namely 1.1 g/day in females and 1.6 g/day in males [77,79,80]. Therefore, the vegan diet, characterized by a low intake of ω-3 fatty acids, should be supported by oral food supplements such as microalgae supplements containing DHA, as well as by a regular consumption of foods with high ALA content in order to guarantee an optimal intake of these fatty acids. A further strategy to ensure adequate intake of ω-3 fatty acids is represented by the consumption of fortified foods such as soy milk and cereals [62].

Numerous studies have focused attention on a possible deficiency of vitamin B12 in vegans, as it is contained in food like meat, eggs, fish, milk, cheese, etc. [67,81]. Vitamin B12 is an essential micronutrient which is involved in numerous biochemical activities such as the maturation of red blood cells, the functioning of the nervous system and the biosynthesis of neurotransmitters [82]. Signs and symptoms of vitamin B12 deficiency are well-known (fatigue, wheezing, lack of energy, headache, irritability, possible anemia, pallor, depression, sleep disorders, and other general impairments) [83]. Vitamin B12 deficiency can occur both in vegetarian and in vegan diets [84,85]. The exclusion of foods containing vitamin B12, can only have an effect after a long time, as the liver reserves guarantee adequate levels of this vitamin for several years [83,86]. Its deficiency is more common in LOV and vegan dietary patterns [84]. All people who follow a vegan diet should supplement it with a reliable source of vitamin B12, through fortified food or oral food supplements, up to 2500 μg per week [67,87]. In a cross-sectional study performed in Germany entitled “The Risks and Benefits of a Vegan Diet”, the authors stated that vitamin B12 status was similar in vegans and omnivores, even though vegans assumed a lower amount [88]. This data can be explained by the high oral supplementation rate in these subjects, according to previous studies [85,87]. In addition to the fortified foods, which are able to counteract vitamin B12 deficiency contained in vegetables, milk, yeast and cereals [89], the market offers oral food supplements namely (i) methyl-cobalamin, (ii) adenosyl-cobalamin, and (iii) hydroxy-cobalamin [90].

The main sources of vitamin D are fish and fish oil, meat from cattle, pigs and poultry and egg yolk. Vitamin D is produced in the human skin after the exposure to ultraviolet B (UVB) rays. Vitamin D is defined as a pro-hormone as it needs to be activated by liver and kidneys. The only non-animal foods that contain significant amounts of vitamin D is the mushroom exposed to the sun or to ultraviolet rays. The most common form of vitamin D in mushroom is vitamin D2, with lower amounts of vitamin D3 and vitamin D4. Although vitamin D2 levels in mushroom may decrease as a result of storage and subsequent cooking, if consumed immediately after harvesting, the vitamin D2 level remains higher than 10 mg/100 g at dry weight [91]. The dietary intake of vitamin D in vegans has been investigated by one of the major epidemiological studies conducted in the United Kingdom [92]. The results demonstrated that levels of vitamin D in the blood were, on average, lower than omnivores and vegetarians; therefore, this data indicates lower reserves and reduced bone mineralization in vegans [93]. In fact, a study conducted by Hansen et al. demonstrated that calcium blood values were significantly lower in vegans compared to omnivores. Although in vegans, the sources of calcium are several (Chinese cabbage, broccoli, turnips, dandelion, watercress, dried figs, sesame seeds, almonds, oranges, tahini, sweet potatoes, beans, bread and cereals), its absorption is limited due to the low serum levels of vitamin D, which prevent adequate calcium gut absorption [93,94]. For this reason, in the presence of inadequate serum levels of vitamin D and calcium, vegans should consume food enriched in vitamin D, such as soy milk, rice milk, and juices [62].

Iron deficit may represent an additional complication in people following a vegan diet. The iron content in PBD is quite similar to that of omnivores, but its bioavailability is lower due to the absence of heme iron. In particular, vegans show a higher iron content than LOVs due to the enhanced consumption of legumes [95]. The main sources of iron in vegans are represented by legumes (beans, lentils, peas, peanuts), leafy greens, soy and its derivatives, quinoa, potatoes, dried fruit, etc. [67]. Studies on iron metabolism conducted by Waldmann et al. have shown that serum ferritin is lower in vegans than in omnivores and that hemoglobin levels are similar or slightly lower in vegans compared to omnivores [96]. A further study conducted by Pawlak et al. stated that in the vegan population, subjects at greatest risk of developing iron deficiencies are menopausal women [97]. Therefore, in case of iron deficiency, it is possible to consume fortified food such as salt, wheat flour and rice [98].

In plant-origin food, there are some factors that induce a low absorption of zinc, such as the abundant presence of phytates [99]. Despite this, in the scientific literature, there are no reports of zinc deficiency in vegans, as its intake is between 7 and 10 mg per day, like in omnivores [88]. As a precaution, some guidelines recommend for vegans and for LOVs a 50% increased intake of zinc compared to omnivores [100]. Foods containing a good amount of zinc are pumpkin seeds, followed by sunflower seeds, nuts and peanuts. Fortified foods in zinc are also available such as cereals [62].

Iodine is essential for the synthesis of thyroid hormones that, in turn, regulate cell metabolism. Iodine deficiency can cause hypothyroidism and goiter, apathy and mental disorders [101,102]. The main sources of iodine in vegans are salt and seaweed. For this reason, vegans can show deficiency of iodine, especially if they live in geographical areas poor in iodine [88,103,104]. Iodine levels in vegans were found to be below the limits set by the World Health Organization, therefore oral food supplements rather than fortified foods such as salt, potatoes, carrots, etc. are strictly recommended in vegans [8,72,105].

## 4. Metabolic Syndrome

**Figure 4:**
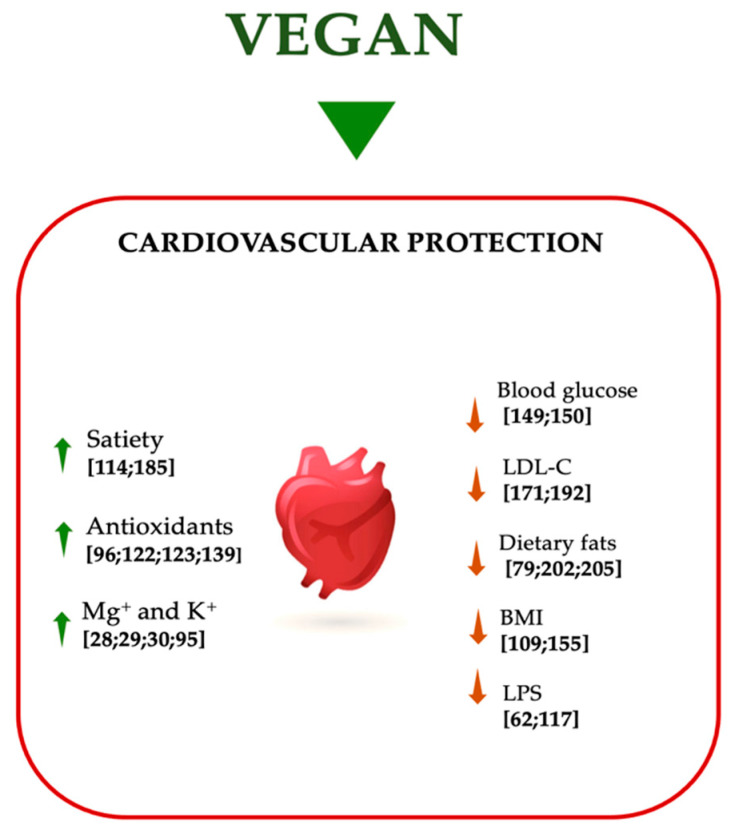
Effects of vegan diet on cardiovascular protection. Abbreviations: BMI, body mass index; K^+^, potassium; LDL-C, low-density lipoprotein-cholesterol; LPS, lipopolysaccharide; Mg^+^, magnesium; ↑ increase; ↓ decrease.

MetS is a clinical condition characterized by a series of metabolic impairments, that cause an increased cardiovascular risk [33,41]. The positive association between PBD and cardiovascular risk is well-known in literature; in fact, people that follow a PBD regimen, and especially vegans, have a lower incidence of CVDs (Figure 4) [85].

In this context, a study conducted by Key et al. on vegetarians highlighted that the death rate for coronary heart disease was 24% lower in subjects that followed a PBD compared to omnivores [106]. In a further meta-analysis conducted by Kwok et al., the mortality rate for stroke was 22% lower in men that followed PBD compared to omnivores, but this association was not significant among women, demonstrating a gender-dependent effect [107,108].

Fontana et al. [109] conducted a study of 21 subjects that followed a vegan diet vs a Western diet, demonstrating that vegan diet was protective against cardiometabolic diseases. In fact, the authors found a reduction in fasting blood glucose and BMI and an improvement in lipid profile. A vegan diet could positively impact on cardiovascular health through a large number of biological mechanisms [29], and on the other hand, on long-term body weight maintenance, compared to omnivores [110].

Vegan diet is characterized by a low energy intake due to its low SFA and high fiber content. Dietary fibers consist of a wide range of plant compounds (carbohydrate polymers with 10 or more monomer units) which vary in their physical and chemical properties [111]. These are typically classified as water-soluble (SFs) and insoluble fibers (IFs). These fibers are not hydrolyzed by digestive enzymes and therefore, they are not fully digested in the human intestine [112]. One of their actions is to influence intestinal function by facilitating food transit time, normalizing intestinal movements, increasing fecal mass and preventing constipation. SF, in particular, dissolve in water and form viscous gels in the intestinal lumen to delay or partially reduce the absorption of carbohydrates, fats and cholesterol. SF is found in vegetables, legumes, fruit such as apples, pears, citrus fruit, and grains such as oats and barley, while IF is found mainly in vegetables, potatoes, nuts and whole grain products such as wheat bran [113].

In particular, when consumed these fibers could reduce energy intake by triggering signs of satiety such as increased gastric distension and the formation of gel due to the absorption of water by SFs. The formation of this viscous gel by SFs can also delay gastric emptying and prolong nutrient uptake, further promoting satiety and modulating insulin and glycemic post-prandial responses [114].

Another key mechanism through which a vegan diet can improve cardiovascular health is its impact on cholesterol metabolism. In fact, the low content of SFAs and the high content of unsaturated fats can improve the lipid profile. In vitro studies have shown that SFAs activate the pro-inflammatory toll like-receptor-4 (TLR4) signaling pathway, which induce the release of cytokines able to trigger a chronic inflammatory status [115,116]. SFAs can also interact with the gut microbiota by promoting the translocation of lipopolysaccharide (LPS), a potent pro-inflammatory endotoxin [57,117]. On the other hand, there are several studies that have shown that polyunsaturated fatty acids (PUFAs) activate several anti-inflammatory pathways. Therefore, a diet enriched in unsaturated fats and low in SFAs can reduce the risk of CVDs through its potential anti-inflammatory effects [118,119].

In addition, we know that vegetable-origin foods are rich in polyphenols, natural bioactive compounds produced by plants as secondary metabolites [120,121]. Polyphenols are classified according to their structure into four large classes: flavonoids, lignans, phenolic acids and stilbenes. Several in vitro studies have shown that polyphenols display a high antioxidant capacity due to their ability in reactive oxygen species (ROS) neutralization [122]. This antioxidant capacity, potentially combined with their ability to modulate the production of nitric oxide (NO), allows polyphenols to preserve endothelial function. Polyphenols could also improve cardiovascular status through the inhibition of platelet aggregation, the reduction of vascular inflammation, the modulation of apoptotic processes, the reduction of LDL-C oxidation and the improvement of lipid profile [123].

Vegan diet is rich in other antioxidant nutrients, such as vitamin C, vitamin E, β-carotene, potassium and magnesium. In particular, it has been shown that potassium is able to reduce BP and the risk of stroke thanks to its beneficial effects on endothelial function and vascular homeostasis [95]. Magnesium has been associated with improved cardiometabolic outcomes for its action on glucose metabolism and on its anti-inflammatory, vasodilatory and antiarrhythmic properties [29].

**Figure 5:**
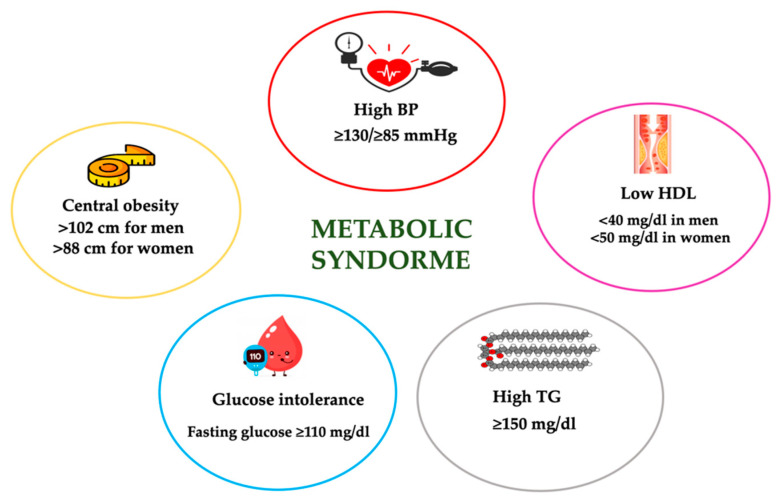
Definition of metabolic syndrome according to Adult Treatment Panel III National Cholesterol Education Program (ATP III-NCEP). BP, blood pressure; HDL-C, high-density lipoprotein- cholesterol; TG, triglycerides.

Among the clinical alterations typical of MetS, it is possible to detect central obesity, dyslipidemia characterized by low HDL-C and high TG, glucose intolerance and T2DM, and high BP (Figure 5). MetS is diagnosed by the presence of at least 3 of the 5 above mentioned criteria, according to the WHO Adult Treatment Panel III National Cholesterol Education Program (ATP III-NCEP) [124]. Moreover, the scientific literature offers further definitions of MetS such as those of the International Diabetes Federation (IDF) [125] and the American Heart Association/National Heart, Lung and Blood Institute (AHA/NHLBI) [126].

The prevalence of MetS, in developed countries, has reached 20–25% with an increasingly enhanced incidence [33], and it increases also with aging [127]. In the elderly, the presence of overweight and/or obesity, T2DM, insulin resistance, arterial hypertension (AH) and alterations in lipid profile is common, partly as a result of changes in body composition, characterized by an increase in fat mass and a decrease in lean mass [128].

Behavioral habits, such as a reduction in physical activity and unhealthy dietary approaches, are linked to an enhancement in insulin resistance, especially if accompanied by a diet with a high caloric intake. Moreover, neurohormonal changes linked to aging, including the reduction of anabolic hormones, such as insulin-like growth factor (IGF)-1 and dehydroepiandrosterone sulfate, and the increase of ROS, (that in turn cause a reduction of antioxidant mechanisms), contribute to the metabolic alterations typical of MetS [129].

**Figure 6:**
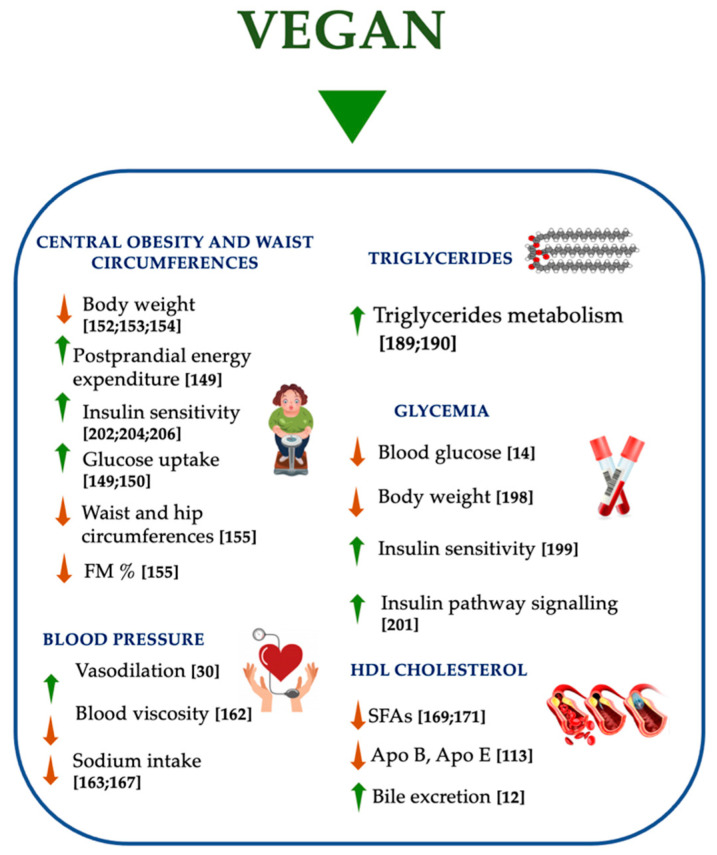
Effects of vegan diet on metabolic syndrome. APO, apolipoprotein; FM, fat mass; SFAs, saturated fatty acids; ↑ increase; ↓ decrease.

In the treatment of MetS, it has been shown that a healthy lifestyle can have a positive effect. PBDs, such as vegan diet, are food patterns useful in the treatment and in the prevention of MetS (Figure 6) [19]. In fact, PBD and vegan diet can play a protective role through different dietary properties: (i) iso/low-caloric intake, (ii) low-SFA level, (iii) high-fibers content, (iv) high intake of fruit and vegetables, (v) elimination or reduction of meat consumption, (vi) no heme iron.

MetS is often accompanied by an excessive body weight [130]. In several randomized studies, the comparison between PBD and omnivorous diets has shown that subjects following a PBD had a lower caloric intake and BMI than omnivores [71]. Several studies have observed that diets characterized by a high presence of SFAs are associated with an increased risk of developing MetS [131,132]. In relation to this, vegan diet and, to a lesser extent, PBDs are characterized by a lower presence of SFAs as demonstrated by observational studies as well as by experimental studies [8,71,133,134]. The American Heart Association and the Institute of Medicine have recommended that adults and children should follow a diet with 10% energy derived from SFAs, in order to maintain a metabolic balance [135,136].

Diets rich in fibers are associated with a lower risk of developing MetS [137,138], because the replacement of an amount of, or all, animal-origin proteins with plant-origin protein sources helps to increase dietary fiber intake. Both observational and experimental studies have shown that fiber intake is significantly higher in PBDs and in vegan diets compared to omnivores [8,71,133,134]. The risk of developing MetS is lower in subjects who follow a diet with a high intake of fruit and vegetables [139] that provides antioxidant and anti-inflammatory substances [139,140].

The consumption of meat, in particular red and processed meat, has been associated with an increased risk of developing MetS, T2DM and cancer [141,142,143]. Several studies demonstrated that subjects who follow a vegan diet appear to have a lower risk of MetS onset [144,145]. Finally, some studies have demonstrated that heme iron, as well as the increase of serum ferritin levels, enhance the risk of developing MetS and T2DM [146,147]. Although heme iron is more absorbable than non-heme iron, most studies do not show that subjects following a PBD present ferritin serum suboptimal levels [64]. Accordingly, the reduced dietary intake of heme iron is another potential strategy to decrease the risk of MetS onset and is another reason that explains why the vegan diet is protective against MetS [148].

### 4.1. Central Obesity and Waist Circumference

PBDs have been shown to be effective in body weight loss, in particular in the reduction of visceral and subfascial fat in the muscle tissue, which in turn is involved in glucose homeostasis [11]. In observational studies conducted by Berkow et al., it has been demonstrated that people who follow PBD typically have a lower body weight than individuals who follow other dietary patterns, suggesting that PBD may be helpful in preventing or treating body composition impairments [61]. In addition, the meta-analysis conducted by Neal et al. showed that PBD prescription is associated with an average body weight reduction of 3.4 kg in an intent-to-treat analysis and 4.6 kg in a comprehensive analysis [149]. In this study, no significant difference was observed in body weight loss between the LOV and people who follow a vegan diet. There are two mechanisms that seem to be involved in the body weight loss associated with diet. The first mechanism is linked to high fiber content, while the second is linked to increased postprandial energy expenditure. In a study of overweight postmenopausal women, a low-fat vegan diet, followed for 14 weeks, induced a significant weight loss related to increased insulin sensitivity by improving glucose cell uptake [149,150]. In a study carried out in 2014 by Huang et al., it was found that subjects who follow a PBD had a marked body weight decrease, compared to an omnivorous diet. In fact, the researchers observed a body weight reduction of 2.52 kg against 1.48 kg of the subjects that follow an ad libitum diet [151].

Another randomized controlled trial conducted by Turner-McGrievy et al. has analyzed the impact of vegan diet on weight loss. This study confirmed that vegan diet promotes weight loss in a statistically significant manner, compared to omnivorous, semi-vegetarian, and FV diets, after a six–month follow up. In fact, vegan diet leads to an improved macronutrients, fiber and cholesterol intake, resulting in a dietary pattern useful in the prevention and treatment of obesity [152]. In a randomized study conducted by Moore et al. in 2015, it was shown that overweight or obese people obtain a greater weight loss following a vegan diet rather than following dietary patterns such as vegetarian, semi-vegetarian and FV diets [153,154].

A study by Chen et al. [155], with a seven years follow-up, evaluated whether a “strict” vegan diet was able to prevent abdominal fat accumulation in aged subjects. The results demonstrated that a higher adherence to vegan diet allowed a lower BMI, waist-hip circumference, and fat mass (in percentage terms), confirming that this diet is associated with a better body composition compared to a free diet. This effect seems to be induced by compounds contained in plant-based products such as fibers, chlorogenic acids, antioxidants, vegetable proteins, and ω-6 fatty acids thanks to the induction of satiety and through the modulation of gut microbiota [156], inflammatory status [157,158] and oxidative stress [159,160,161].

Long-term intervention studies are needed to understand the effect of these dietary patterns on body weight control and loss.

### 4.2. Blood Pressure

High BP is the most important cardiovascular modifiable risk factor [162], accounting for about 50% of all heart and brain ischemic events [32,163].

Dietary patterns play a fundamental role in the prevention and treatment of AH. Millions of individuals worldwide suffer from AH [162,164], which is defined as SBP of over 140 mm Hg and DBP of over 90 mm Hg. The onset of high BP can be delayed and counteracted with the adoption of a proper diet. The latter should be characterized by a low SFAs intake, a high fruit and vegetables intake, reducing the amount of food rich in salt (added or naturally present), and limiting the consumption of alcohol to achieve and maintain over time the correct body weight.

In fact, the positive mechanisms on AH are a better vasodilation, an increased intake of natural antioxidants and anti-inflammatory compounds, an improved insulin sensitivity, a lower blood viscosity and a positive change in gut microbiota composition [165,166].

Some dietary patterns and food groups are associated with a lower risk of developing AH. PBDs are associated with lower BP levels, compared to those of omnivores, and they can reduce SBP by 6.7 mm Hg and DBP by 5.9 mm Hg [167,168]. On the contrary, red and processed meats seem to increase the risk of developing AH; however, this association has not been consistently demonstrated [169].

Vegan diet has not been associated with a significant change in SBP or DBP compared to PBDs that are less restrictive [170]. However, it is interesting to note that in the study conducted by Lopez et al. [170] the basal SBP was 130 mm Hg, and after a vegan diet the authors observed an average decrease of 4 mm Hg in both SBP and DBP. In this study, it was also demonstrated that the effect of vegan diet on BP is similar to that of diets recommended by medical societies [167,171]. This dietary pattern is characterized by a reduced dietary sodium intake, and lower SBP from 1 to 4 mm Hg in normotensive individuals, and from 5 to 7–8 mm Hg in AH patients. Further randomized clinical trials are necessary to assess the role of a vegan diet on BP control in hypertensive patients.

### 4.3. Lipid Metabolism

The beneficial effects of food components of plant origin directly or indirectly entail a lower risk of developing CVDs and influence several metabolic pathways, such as lipid metabolism [12,172,173]. The fat quality is determined by the content of different kinds of fatty acids. Animal fat such as meat, butter, whole dairy products, as well as tropical coconut oil and palm oil, are typically rich in SFAs. On the contrary, vegetable fats, consisting mainly of vegetable oils, are generally rich in unsaturated fatty acids. The latter can be monounsaturated (MUFAs), such as oleic, or PUFAs. The substitution of SFA with unsaturated fatty acids reduces LDL-C without affecting HDL-C and TG. The effect on LDL-C reduction is greater when SFAs are replaced with PUFAs [174]. Substituting for SFAs with carbohydrates induces a lowering of LDL-C and HDL-C but causes an increase in fasting TG. Therefore, it does not improve the overall lipid profile [175]. Therefore, to obtain the best beneficial effect on lipid metabolism, it is necessary to replace SFAs with unsaturated fats. The intake of PUFA as very long chain ω-3 (EPA and DHA) in vegan diet is allowed by fish oil intake (if necessary, also as oral food supplement) and it has no substantial effect on LDL-C, while affecting the concentrations of TG, that are lower in a dose-dependent manner [80,113,176]. For this reason, a PBD for HDL-C control seems to be more effective. In addition, vegan diets are rich in dietary fibers and it has been shown that an intake of 4–10 g/day of different types of SFs results in a reduction of 5–10% of LDL-C without influencing the values of HDL-C and TG. In fact, SFs have an high viscosity and decrease the absorption of macronutrients, cholesterol and bile acids, causing an increased fecal excretion [177]. Reduced resorption and increased excretion of bile acids stimulate their synthesis in the liver which, in turn, reduces serum TC concentration.

PBDs are also characterized by the presence of phytosterols (PSs) including plant sterols and stanols, which are similar to cholesterol both in structure and function. Cholesterol is an organic compound that plays important functions in the body, including the synthesis of numerous hormones and the formation of cell membranes, in particular of the nervous cells. It is produced endogenously in the body, namely in the liver, and it is absorbed exogenously through food. Cholesterol is present in all animal-origin food (red meat, poultry, eggs, milk, cheese), while it is not present in any vegetable-origin food [178]. HDL-C also displays a protective effect on atherosclerotic diseases [179].

PSs are naturally found in all plant-based foods and in vegetable oils (especially unrefined oils), in margarines based on vegetable oil, in seeds, nuts, cereals, legumes, vegetables and fruit [180]. A 2 g/day intake of PSs reduces the cholesterol absorption by 30–40%, resulting in a 10% reduction in circulating LDL-C [181]. The mechanisms of action are multiple: (i) partial inhibition of intestinal absorption of cholesterol (dietary and biliary), (ii) transfer of cholesterol from mixed micelles due to the limited ability to incorporate sterols and (iii) stimulation of cholesterol excretion through transintestinal via. In addition, it has been shown that the intake of PSs is able to reduce the atherogenic apo-lipoproteins such as Apo-B and Apo-E and to increase the anti-atherogenic apolipoproteins such as Apo-AI and Apo-CII [113].

Serum concentration of TG and the HDL-C to TC ratio tend to be lower in people who consume PBDs [13]. Several studies demonstrated that PBDs are able to lower plasma lipids, in particular TGs, compared to omnivores [182,183,184,185,186]. In this regard, subjects who follow a PBD tend to show lower LDL-C values than omnivores [187,188]. In a three-group randomized crossover study performed in 60 subjects that followed a PBD, this dietary model increased postprandial secretion of gastrointestinal hormones, as well as promoting satiety, compared to diets with processed meat and cheese, both in healthy, obese, and diabetic men [189]. Thus, these properties could have practical implications for the management of hypertriglyceridemia in some metabolic conditions. In a cross-sectional study conducted in Slovenia, participants were instructed to follow a PBD-based program divided into (i) short, (ii) medium and (iii) long term intervention. In particular, the dietary intervention lasted between 0.5 and 10 years. Women who followed the PBD for a longer period showed significantly lower plasma TG and LDL-C values compared with others [190]. Another systematic review demonstrated that PBD is associated with lower plasma lipids. In particular, the authors found decreased values of TC, LDL-C, and HDL-C, but not of plasma TGs [191]. Furthermore, an 8-week PBD program, that strictly excluded animal-based foods and minimized processed foods, did not significantly reduce TG. In this study-intervention, PBD caused an increase of TG, but it was not statistically significant [192].

In the study conducted by Vinagre et al. [193], the authors showed that vegans have a better regulation of lipid metabolism, in particular that of TG [194]. Vegan diet is more efficient in removing potentially atherogenic residues [195].

It has been demonstrated that vegan diet improves metabolic pathway of TG-rich lipoproteins since the removal of remnant lipoproteins from circulation is faster in vegans compared with omnivores, while the lipolysis process seems to be equal [193]. According to a meta-analysis conducted by Benatar et al. which investigated one or more cardiometabolic risk factors in vegans vs omnivores (control group), TG and other lipid parameters (such as LDL-C) were lower in vegans compared to omnivores [196].

Dietary fiber is inversely related to TG levels, as demonstrated by Li et al. in a randomized cross-over trial on 21 healthy participants (vegans vs. omnivores) in which vegans had a lowering of TG levels after 3 days of dietary treatment [197]. Currently, the debate regarding the usefulness of vegan diet on plasma TG control remains unsolved.

### 4.4. Glycemia

The prevalence of T2DM appears to be relatively low among individuals who follow PBDs [198]. Numerous clinical studies have shown an improvement in the glycemic control and in the reduction of CVD onset [14]. A 12-weeks pilot trial, conducted on T2DM patients who followed a vegan diet, showed a 28% decrease of blood glucose levels, compared to the 12% measured in the control group that instead followed a free diet [14]. The benefits of the PBD on the risk of T2DM onset are attributable not only to the abolishment of meat but also to its richness in plant-based foods [199]. Strict adherence to PBD offers more benefits in body weight and glycemic control than a diet based on the nutritional recommendations of the American Diabetes Society (ADA) and/or the European Society for the Study of Diabetes (EASD) and it is associated with a greater reduction in the dose of hypoglycemic drugs [199].

This PBD long-term sustainability is probably due to the wide variety of food availability, its high palatability and its increased satiating effect. Of course, PBD represent a particular dietary pattern that cannot easily be proposed to most T2DM patients in the Western world because the possible high intake of carbohydrates, induced by PBD, needs to be supervised by a nutritionist through a personalized dietetic plan [199]. Intervention studies that evaluated the effectiveness of PBD on glycemic control in T2DM patients are few and have a very limited number of participants [198,199,200]. Moreover, the greatest effects are observed with calory restricted diets that lead to a body weight loss, a factor well-known to influence glycemic control [201]. On the basis of these considerations, ADA states that, in order to clarify the effectiveness of PBD on glyco-metabolic control, further studies are needed to assess the quality of the diet, since the available studies are more focused on food that is not consumed rather than on that consumed [202].

Food to be preferred and consumed for the prevention and treatment of diabetes mellitus certainly contains a greater number of fibers. For this reason, plant-based food is the only food that guarantees the supply of these nutrients. Soya is a common protein replacement for subjects who adopt PBD regimes, since it contains high levels of lysine, leucine, isoleucine, phenylalanine, calcium and phosphate, which have been shown to help glycemic control and insulin sensitivity [203,204].

The intake of cereals, especially whole grains, reduces the risk of diabetes mellitus onset as they are rich in magnesium and fibers. Consuming sufficient quantities of magnesium is essential because its deficiency impairs insulin pathway signaling [205].

A randomized clinical study, conducted in 2005 by Barnard et al., evaluated the efficiency of the vegan diet in patients with T2DM. The subjects were divided into two subgroups, where the first adopted a low-fat vegan diet, and the second a vegan diet according to the ADA guidelines. Both groups improved their glycemic and lipid profile, but these results were more marked in the group that followed a low-fat vegan diet [206].

Several studies have shown that vegan diet has a protective role against T2DM and obesity [24,207]. In fact, a study conducted by Agrawal et al. on an adult Indian population showed that vegans have a lower prevalence of T2DM and obesity than omnivores [208].

These beneficial effects are due to the absence of animal-origin fats and to the increased consumption of food with a low glycemic index [209]. Among these are whole grains that improve insulin response and reduce the risk of developing T2DM through the action of nutrients such as vitamin E and magnesium [210].

## 5. Conclusions

PBDs, in particular the vegan diets, represent a food pattern adopted for years by groups of people, primarily on the basis of ethical, ideological and environmental reasons. Currently, a vegan diet is mostly adopted in order to improve body weight and body composition, as well as the typical alteration of MetS [11]. Accordingly, this dietary pattern seems to be useful in the prevention and treatment of MetS and CVDs if well-planned by a nutritionist. For this reason, long-term clinical studies should be carried out in order to define the impact of the vegan diet on chronic non-communicable degenerative diseases onset and progression, such as MetS and CVDs.

## Figures and Tables

**Table 1:** Examples of plant-based dietary patterns. FV, fish-vegetarian; LOV, lacto-ovo-vegetarian; PBD, plant-based diet.

Dietary Approach	Model	Characteristics of Dietary Patterns
PBD	VEGAN	Does not contain any animal products (meat, fish, poultry, eggs, or dairy products) but emphasizes plant-based foods, like fruit, vegetables, whole grains, legumes/beans, nuts and seeds.
	LOV	Plant based foods, such as fruit, vegetables, whole grains, legumes/beans, cheese and various dairy products.
	FV	Plant-based foods, such as fruit, vegetables, whole grains, legumes/beans, fish and seafoods.

## Data Availability

Not applicable.

## Abbreviations

ADA	American Diabetes Society
AH	Arterial Hypertension
AHA/NHLBI	American Heart Association/National Heart, Lung and Blood Institute
AI	Adequate Intake
ALA	α-Linolenic Acid
ATP III-NCEP	Adult Treatment Panel III—National Cholesterol Education Program
BMI	Body Mass Index
BP	Blood Pressure
CVD	Cardio-Vascular Disease
DASH	Dietary Approaches to Stop Hypertension
DBP	Diastolic Blood Pressure
DHA	Docosahexaenoic Acid
EASD	European Society for The Study of Diabetes
EPA	Eicosapentaenoic Acid
EVOO	Extra-Virgin Olive Oil
FAO	Food and Agriculture Organization of The United Nations
FV	Fish-Vegetarian
GHG	Green House Gas
HDL-C	High-Density Lipoprotein-Cholesterol
IDF	International Diabetes Federation
IF	Insoluble Fiber
IGF-1	Insulin-like Growth Factor-1
LDL-C	Low-Density Lipoprotein- Cholesterol
LOV	Lacto-Ovo-Vegetarian
LPS	Lipopolysaccharide
MD	Mediterranean Diet
MetS	Metabolic Syndrome
MUFA	Mono-Unsaturated Fatty Acid
NO	Nitric Oxide
PBD	Plant-Based Diet
PS	Phytosterol
PUFA	Poly-Unsaturated Fatty Acid
ROS	Reactive Oxygen Species
SBP	Systolic Blood Pressure
SF	Water-Soluble Fiber
SFA	Saturated Fatty Acid
T2DM	Type 2 Diabetes Mellitus
TC	Total Cholesterol
TG	Triglyceride
TLR4	Toll-like Receptor 4
TNF- α	Tumor Necrosis Factor-α
